# The Role of β1 Integrin/CD29 as a Potential Prognostic Factor for the Risk of Progression to Cervical Carcinoma in HPV-Associated Lesions

**DOI:** 10.3390/medicina60030364

**Published:** 2024-02-21

**Authors:** Maria Teresa Schettino, Eleonora Petra Preti, Valeria Vietri, Nadia Agrillo, Nicola Iavazzo, Diego Domenico Fasulo, Pasquale De Franciscis, Maria Rosaria Campitiello, Maria Giovanna Vastarella, Gaetano Riemma, Barbara Gardella, Filippo Murina

**Affiliations:** 1 Department of Woman, Child and General and Specialized Surgery, University of Campania “Luigi Vanvitelli”, 80138 Naples, Italy; mariateresa.sche@gmail.com (M.T.S.); valevietri@tiscali.it (V.V.); agrillo.nadia@hsr.it (N.A.); nicolaiavazzo@live.it (N.I.); diegodomenico1993@gmail.com (D.D.F.); pasquale.defranciscis@unicampania.it (P.D.F.); 2 Preventive Gynecology Unit, European Institute of Oncology, 20100 Milano, Italy; eleonora.preti@ieo.it; 3 ASL Salerno, 84100 Salerno, Italy; mr.campitiello@sanita.it; 4 Department of Obstetrics and Gynecology, Fondazione IRCCS Policlinico San Matteo, 27100 Pavia, Italy; barbara.gardella@gmail.com; 5 Lower Genital Tract Disease Unit, Obstetrics and Gynecology Department, V. Buzzi Hospital, University of Milan, 20154 Milan, Italy; filippo.murina@unimi.it

**Keywords:** HPV, integrins, cervical intraepithelial lesion, CD29, β1 integrin, colposcopy

## Abstract

*Background and Objectives*: Available evidence reports the overexpression of β1 integrin in dysplastic rather than normal cervical tissue. We aimed to evaluate the involvement of β1 (CD29) integrin in the progressive pathogenesis of cervical intraepithelial neoplasia (CIN). *Materials and Methods*: From January 2019 to December 2021, we prospectively enrolled women undergoing a colposcopy with a cervical biopsy for abnormal cervical cytology and/or undefined cytology with a positive HPV DNA test and women with relapsing cervical inflammatory disorders. Based on the histopathological results, women were divided into four groups: group A (CIN1), group B (CIN2), group C (CIN3), and group D (no CIN diagnosis) as a control group. Subsequently, cytofluorimetry and immunohistochemical analysis (based on the identified positive cell ratios as follows: ≤10%, negative; 10–25%, 1+ (weak); 25–50%, 2+ (medium); ≥50%, and 3+ (high)) for β1 integrin were carried out. *Results*: In total, 154 women were included. The average fluorescence intensity in the four groups was 2.35 ± 1.37, 2.73 ± 1.56, 3.09 ± 1.56, and 2.13 ± 1.25 UA from groups A to D, respectively; this figure was significantly different for CIN3 (group C) women relative to the other groups (*p* = 0.0132). Higher β1 integrin/CD29 concentrations in the CIN groups with HR-HPV 16 and 18 were also detected (*p* = 0.0292, 0.0367, and 0.0357 respectively for CIN3, CIN2, and CIN1). Immunohistochemistry analysis showed higher results for the CIN3 group compared to controls and all the other groups (*p* < 0.001). *Conclusions*: β1/CD29 integrin expression increased with CIN grade, and it was significantly higher in CIN3 lesions. This could be used as a promising screening tool to identify women prone to developing high-grade cervical lesions. However, additional evidence is needed to strengthen these findings.

## 1. Introduction

Integrins are a family of transmembrane receptor proteins involved in complex interactions between the extracellular matrix (ECM) and cell cytoskeleton structure. Among these, exosomes—40–100 nm diameter spherical or cup-shaped bubbles—are released by numerous cells and present in all biological fluids. Numerous cellular proteins, nucleic acids, mRNA, miRNA, and DNA as well as soluble components including chemokines and cytokines, enzymes, and cofactors are all present in the exosome lumen. Different patterns of integrin expression were identified by exosome proteomics and related to metastasis development. In fact, targeting the α6β4 and αvβ5 integrins reduced the uptake of exosomes and the metastasis of lung and liver cancer, respectively [1]. Therefore, two-way integrin signaling ensures responses both on the intracellular and the extracellular sides [2,3]. Cells rearrange the cytoskeleton structure when the extracellular integrin domain binds ECM proteins; it is also rearranged by the activation and/or exposure of new binding sites following intracellular input due to conformational changes of integrins in the intracellular domain [3]. Therefore, integrins have a key role not only in cell adhesion to ECM proteins inside intercellular spaces and to the basal membrane but also in cellular migration and survival via intracellular signal transduction [4]. Moreover, by enhancing cyclin expression, integrins mediate entry into the cell cycle and promote cellular proliferation [5].

The normal tissue architecture reflects a homeostatic balance between key receptors that rule cell–ECM interactions. This balance is disrupted in cancer tissue, where receptors and ligands are abnormally expressed [6]. The growing importance of cell–ECM interactions in tumor microenvironments for cancer development has brought to attention new biomarkers and molecular targets for therapeutic purposes [7].

Most of the classic literature involved the integrins family in tumor cell proliferation, migration, and survival, and gave it a central role in the invasive and metastatic processes.

In particular, β1 integrin, also known as CD29, has been extensively investigated in the biology of solid tumors due to its ascertained involvement in the proliferation and differentiation of normal epithelial cells.

Its expression has been linked to poor prognoses in lung and pancreatic tumors and cutaneous melanoma. In human breast cancer, β-1 integrin has been involved in tumor progression and metastasis. In fact, it has been proven that beta-1 integrin signal enhancement can gain tumorigenesis by increasing growth factor receptor (GFR) activity [8,9].

Based on this evidence, β1 integrin has become an interesting target for immunotherapy in different subtypes of cancer [10,11].

Globally, cervical cancer is the fourth most common neoplastic pathology in women, and human papillomavirus (HPV) is known to be the cause of the disease, even with vaccination laws and screening programs in place in many nations. In 1988, the National Health Service of England launched a nationwide cervical screening program with the goal of reducing the disease’s burden. Since then, the program has significantly decreased the number of cases in England by more than one-third. Since cervical cancer is uncommon in younger people, screening for the condition is available starting around the age of 25 [12,13]. It is recommended that established cytology-based programs progressively incorporate more HPV DNA testing in order to increase their effectiveness and safely extend the screening interval. With the exception of severely resource-constrained programs that can only afford to use visual inspection-based screening, HPV DNA testing should always be the primary screening test in newly deployed programs due to its superior sensitivity when compared to cytology [12,14].

The underlying molecular mechanisms of HPV-induced carcinogenesis are not fully understood, though, due to their high level of complexity. Cervical cancer cannot develop without persistent HPV infection, but this requirement is insufficient. A favorable post-infection microenvironment (PIM), which is increasingly understood to be essential for viral persistence, multiplication, and malignant progression, is actively shaped by HPV-infected cells in the surrounding environment. A complicated interaction between immune cells, host stroma, and virus-infected cells as well as their derived components like chemokines, cytokines, extracellular vesicles, and metabolites initiates and establishes the PIM [15].

It has been shown that in squamous cervical cancer, β1 integrin expression is significantly higher than in normal cervical tissues and increases with clinical stage and malignity grade [16].

A study conducted at our institution in 2020 analyzed samples of cervical tissue with histological diagnoses of various cervical intraepithelial neoplasia (CIN) grades [17]. It showed significant expression of some markers typically expressed both in mesenchymal stromal cells (MSC), such as CD29 and HLA-I, and hemopoietic cell lines, such as CD38, CD31, CD34, and HLA-II. It has been observed that the expression of CD38, CD29, and HLA-II cell markers increases with the increase of cell dysplasia grade, and there is a higher expression of cell marker CD10 in cervical intraepithelial neoplasia (CIN)3 samples, rather than in CIN1 ones [17]. This expression trend suggests a clinical utility of these markers to define lesions as being at high risk of neoplastic progression [17].

Therefore, based on the available literature and the results of the aforementioned study, we questioned the involvement of β1-CD29 integrin in the evolutionary history of squamous pre-cancerous lesions of the cervix and their dysplastic process, which, from normal histological pictures, leads to micro-invasive squamous carcinoma if no treatment occurs.

## 2. Materials and Methods

From January 2019 to December 2021, we prospectively enrolled women undergoing a colposcopy with a cervical biopsy at the outpatient service for cervical–vaginal pathology of the Obstetrics and Gynecology Unit of a tertiary care university hospital (AOU Luigi Vanvitelli, Naples, Italy). The inclusion criteria involved women aged between 27 and 35 years old who were not vaccinated for HPV or nulliparous and for whom it was necessary to proceed to a targeted cervical biopsy after a colposcopic view had been carried out for undefined or abnormal cervical cytology and a positive HPV DNA test. We also enrolled in the study women who matched the previous criteria and who had a colposcopic view suggestive of extensive squamous metaplasia caused by chronic-relapsing cervical inflammatory disorders, associated with severe clinical symptoms, which required a histopathological diagnosis to exclude invasive lesions that required laser vaporization surgery.

Exclusion criteria were positive anamnesis for autoimmune diseases and/or oncologic diseases; any previous cervical surgery; cervical and vaginal swabs positive for *Chlamydia*, *Ureaplasma*, *Mycoplasma*, *Neisseria*, *Trichomonas* species infections [18]; and a confirmed serologic diagnosis of *Syphilis* and/or a human immunodeficiency virus (HIV) infection. Women were also excluded in case of unsuitability for the biopsy, decline of the procedure, refusal of the procedure, and not signing a written informed consent form.

Histopathological diagnoses of CIN1, 2, and 3, for human cervical tissue were obtained by squamous–columnar junction (SCJ) and congenital transformation zone (CTZ) colposcopic-targeted biopsies.

Based on the histopathological results, patients were divided into four groups: group A, women with a diagnosis of CIN1; group B, women with a diagnosis of CIN2; group C, women with a diagnosis of CIN3; and group D, which included women with a diagnosis of squamous metaplasia caused by cervical inflammatory disorders used as a negative (with no CIN diagnosis) control group.

All the cervical tissue samples were washed twice in PBS (Dulbecco’s Phosphate buffer saline; Microgem, Naples, Italy); next, they were cut into 1 mm pieces and left to float in DMEM (Dulbecco’s Modified Eagle Medium; Gibco, Thermo Fisher Scientific, Waltham, MA, USA) with Aining 2% FBS (Fetal Bovine Serum; Gibco, Thermo Fisher Scientific, Waltham, MA, USA), type 1 collagenase (200 U/mL; B ochrom, Berlin, Germany), and type 1 Deoxyribonuclease (DN-25, 50 microg/mL; Merck/Sigma-Aldrich, Darmstadt, Germany).

Tissues were then digested at room temperature by continuous stirring (300 time/min) and dispersed gently by pipetting for 3 min. The obtained digested products were finally washed twice in 0.15 mM PBS (pH 7.4) and suspended again in 1 mL PBS, ready to be marked with monoclonal antibodies.

### 2.1. Cytofluorimetry

The cytofluorimetric analysis was carried out directly after the abovementioned procedures and according to international guidelines. Ten different tubes (including a negative control) were prepared for each patient. In every tube, the monoclonal antibody was diluted by 1:10 in 100 μL of 1 × 10^6^ cells and incubated for 30 min at 4 °C. After the incubation, they were pelleted and washed twice in PBS and replaced in 400 μL. The mouse-human monoclonal antibody (MoAb) used for flow cytometric immunophenotyping was CD29 (clone MAR4—cod:555443).

The Phycoerythrin fluorochrome (PE) conjugated to the monoclonal antibody CD29 was used for direct immunofluorescence.

The fluorescence intensity (F.I.) was expressed in logarithmic arbitrary units (A.Us.) for ease of data analysis and graphic reproducibility.

The direct proportionality relationship between F.I. and the fluorochrome quantity that produced it guaranteed a quantitative evaluation of binding sites useful for the monoclonal antibody–fluorochrome complex in every cell, and so it allowed for an indirect analysis of specific cell antigens (β1 integrin/CD29) present in the tested sample.

Cells were consecutively analyzed by means of flow cytometry (FACSCanto TMII; Becton Dickinson, Franklin Lakes, NJ, USA) using acquiring and analysis software DIVA v9.0 (Becton Dickinson, Franklin Lakes, NJ, USA). Becton Dickinson Simultest γ1/γ2a IsotypeControl (IgG1 FITC/IgG2a PE) (cod: 342409) (Becton Dickinson, Franklin Lakes, NJ, USA) was instead used as a negative control.

### 2.2. Immunohistochemistry

CD29 expression detection in the tested cervical biopsy samples was conducted using an anti-CD29 antibody (ab183666 Abcam) (1:100) with the UltraView Universal RED Detection Kit (Ventana Benchmark XT, Roche Diagnostics, Basel, Switzerland).

The samples were considered positive for β1 integrin/CD29 if we observed cells with pointed brown granules in the cytoplasm or cell membrane and if the proportion of positive cells was at least >10%. Five fields were randomly selected and high-power scanned (power, ×400) to calculate the average ratio of positive cells. Based on the identified positive cells ratio, the samples were classified as follows: ≤10%, negative; 10–25%, 1+ (weak); 25–50%, 2+ (medium); ≥50%, 3+ (high).

### 2.3. HPV-DNA Test

The presence of high-risk HPV (HR-HPV) (for HPV 16 and HPV 18) DNA was evaluated by a linear array test (Cobas HPV test, Roche Molecular Diagnostics, Milan, Italy).

The Committee on Publication Ethics (COPE) recommendations (http://publicationethics.org/, accessed on 14 December 2022) and the Helsinki Declaration of the World Medical Association were all adhered to throughout the stages of design, analysis, and data interpretation as well as drafting and modification.

Each participant in this study gave their written agreement to engage in the research and allow data collection and analysis for academic purposes. They also received information about the procedures used in the investigation. Any information that could be used to formally identify the patient was removed from the data using anonymization.

The University of Campania “Luigi Vanvitelli” Ethical Committee released its approval for this study with number #2013918.

### 2.4. Statistical Analysis

All the clinical data were included in a database processed through Microsoft Excel and expressed as average ± standard deviation (SD). Regarding cytofluorimetry, for all four groups, first, the average intensity of fluorescence produced by the analysis of all the samples was calculated; next, using the ANOVA model, the differences between the obtained averages were analyzed. Then, a *t*-test was used to compare, in every group, the average intensity of fluorescence in samples with and without HR-HPV. Practically, in this way, an indirect statistical evaluation of β1 integrin/CD29 in different quantities in the samples with and without CIN and in the samples with and without HR HPV was obtained. The ZEN 2.5 blue pro software (Carl-Zeiss AG, Oberkochen, Germany) was used for immunohistochemistry. Using the chi-square test, we compared the samples’ percentages with the significant positivity of β1 integrin/CD29 in every tested group. A *p*-value (*p*) < 0.05 was considered statistically significant.

Statistical analyses and scientific graphing were carried out using the Statistical Package for Social Sciences (SPSS 24.0, IBM, Armonk, NY, USA).

## 3. Results

In total, 194 women were initially selected. After the application of the exclusion criteria, 40 women were discarded. Therefore, 154 women were included. Based on the cervical histopathological results, they were divided as follows: group A (CIN1), 55 women (35.7%); group B (CIN2), 27 women (17.5%); group C (CIN3), 30 women (19.5%); group D (controls), 42 women (27.3%).

### 3.1. Cytofluorometry

**Figure 1:**
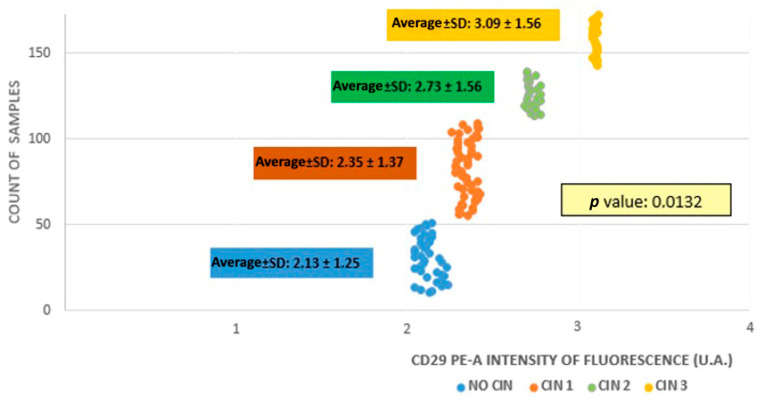
Fluorescence intensity among study groups.

The average fluorescence intensity calculated for the four examined samples was 2.35 ± 1.37, 2.73 ± 1.56, 3.09 ± 1.56, and 2.13 ± 1.25 UA from groups A to D, respectively. As the CIN grade increased, there was a gradual augmentation in the average fluorescence intensity (Figure 1), which meant that there was a higher β1 integrin/CD29 concentration in CIN3 samples compared with CIN1 and CIN2 samples or samples without CIN (*p* = 0.0132).

**Figure 2:**
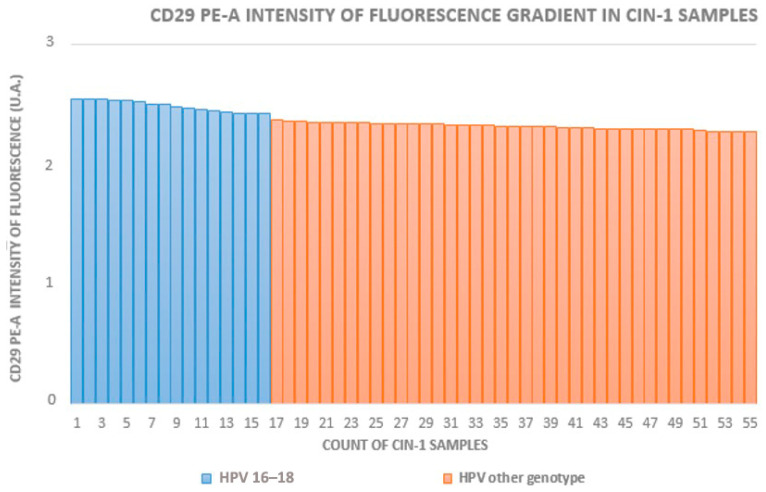
Intensity of fluorescence gradient in CIN1 samples.

**Figure 3:**
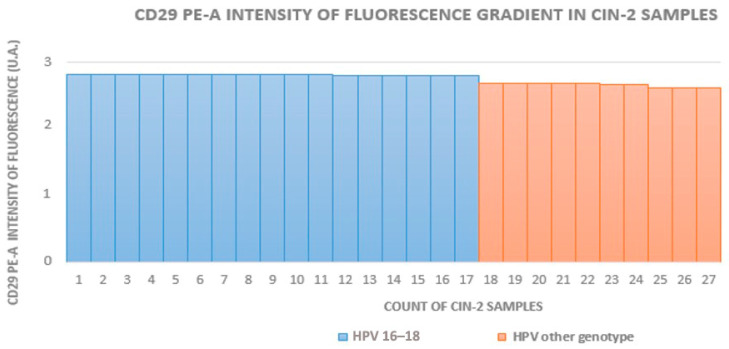
Intensity of fluorescence gradient in CIN2 samples.

**Figure 4:**
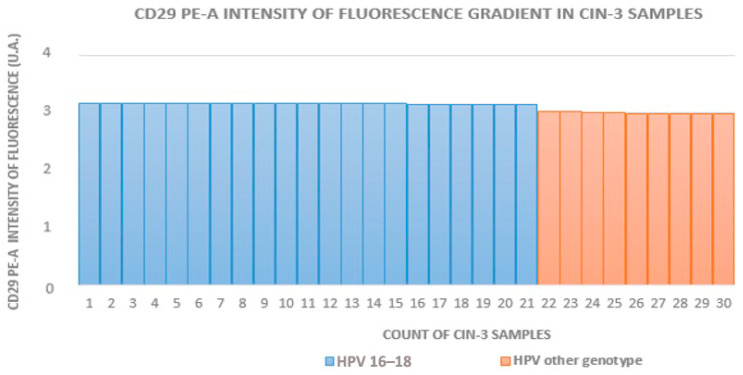
Intensity of fluorescence gradient in CIN3 samples.

The HR-HPV 16- and 18-positive sample rates in groups A to D (which included HPV+ squamous metaplasia) were 29% (16/55), 63% (17/27), 70% (21/30), and 12% (5/42), respectively. In groups with equivalent CIN grades, the fluorescence intensity was higher in HR-HPV 16- and 18-positive samples. Figure 2, Figure 3 and Figure 4 show the distribution of fluorescence intensity values along an increasing gradient obtained in the cytofluorometry analysis of the examined samples of CIN1, CIN2, and CIN3, respectively.

In group A, the average fluorescence intensity in the HR-HPV 16- and 18-positive samples was 2.51 ± 0.79 U.A. and, in the negative samples, it was 2.32 ± 1.18 U.A. In group B, the average fluorescence intensity in the HR-HPV 16- and 18-positive samples was 2.81 ± 1.24 U.A. and, in the negative samples, it was 2.65 ± 0.78 U.A. In group C, the average fluorescence intensity in the HR-HPV 16- and 18-positive samples was 3.22 ± 1.15 U.A. and, in the negative samples, it was 2.97 ± 0.97 U.A.

**Figure 5:**
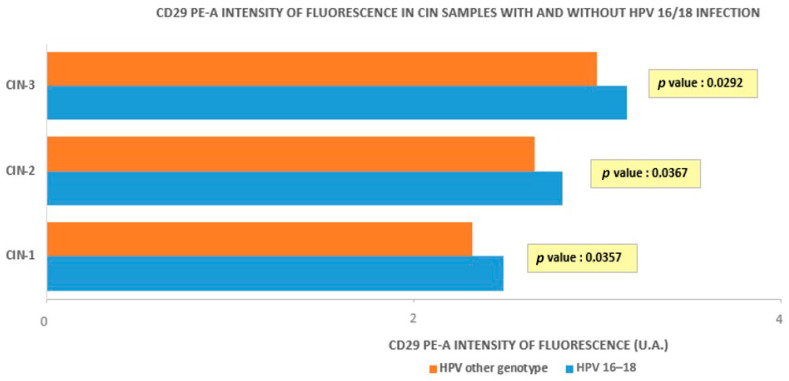
Intensity of fluorescence in CIN samples with and without HPV 16/18 infection.

Figure 5 compares the average fluorescence intensity in HR-HPV 16- and 18-positive or -negative samples, which was higher in the positive ones, according to a higher β1 integrin/CD29 concentration in the CIN groups where HR-HPV 16- and 18-were detected (*p* = 0.0292, 0.0367, and 0.0357, respectively, for CIN3, CIN2, and CIN1).

### 3.2. Immunohistochemistry

Table 1 shows the immunohistochemistry analysis of every group of women according to the criteria outlined in the Materials and Methods section.

In total, 34.5% (19/55) of group A (CIN1) samples showed significant β1 integrin/CD29 expression (>10%).

For CIN2 diagnoses, 18 samples out of 27 (66.6%) expressed β1 integrin/CD29; 86.7% (26/30) of group C samples (CIN3) showed significant β1 integrin/CD29 expression (>10%).

On the other hand, only 7 out of 42 (16.6%) of group D samples (squamous cervical metaplasia caused by chronic inflammatory disease) showed middle-to-low β1 integrin/CD29 expression, which was significantly lower compared with group C (CIN3). The expression of β1 integrin/CD29 was, therefore, higher in the CIN3 group compared to all the other groups (*p* < 0.001).

## 4. Discussion

Our study revealed that β1/CD29 integrin concentration is higher in CIN3 cervical samples and becomes higher with the progress of the CIN stage, reflecting the pre-neoplastic evolution of the cervical intraepithelial lesion.

Many studies whose aim was to analyze the integrins chain in the cervical epithelium confirmed the fine modulation in the dysplasia’s progression. The most common change is the suprabasal integrin expression, which seems to be typical of early dysplasia associated with hyperproliferative input and lower differentiation [19]. Previous studies of the cervical epithelium’s dysplasia show a wide range of the expression of the integrin in different areas of the same sample and in samples at the same CIN stage [19].

So far, the exact immunohistochemical localization of the integrins chains in the dysplastic cervical epithelium has not provided coherent criteria for an exact evaluation of the disease’s stage [20].

Therefore, from a clinical point of view, considering the increasing expression of the β1/CD29 integrin during active HPV infection, we could consider monoclonal antibody anti-CD29 as a promising reagent for early stratification and during the risk triage of the patient having or developing an advanced-stage cervical lesion.

Ultrastructural studies show integrin chains’ distribution in the basal cell layer of the squamous epithelium, which is crucial for the interaction between the epithelium and basal lamina [20]. The interactions between them can be altered during the first steps of dysplastic transformations that come before micro-invasive cervical cancer [21,22,23].

As a matter of fact, altered integrin expression has been observed in a variety of hyperproliferative skin conditions (for example, psoriasis and/or benign tumors like basal cell carcinoma) and dysplastic conditions (for example, actinic keratoses and invasive stages of squamous cell carcinoma) [24].

In hyperproliferative skin conditions, integrins are also expressed in the suprabasal layer or highly expressed in all tumoral cells; in dysplastic conditions, some integrin chains, like β1 integrin, are retained, while others, like α6β4 integrins, show an altered distribution pattern [25,26].

Analyzing cervical samples at the same CIN stage, we found the overexpression of CD29 in samples associated with positive HPV tests for HR-HPV 16 and 18 strains. This reinforces the potential clinical applicability of the β1-CD29 integrin as an accurate prognostic biomarker in pre-invasive cervical lesions; in fact, this system can identify patients with a high risk of developing high-grade/CIN3 lesions, the true precursor of micro-invasive cervical cancer. However, due to the lack of analysis of other subtypes of HR HPV (excluding HPV 16 and 18), such a characteristic could not be linked to all the HR genotypes. Even if HPV 16 was associated with CIN2 persistence in about 70% of the women, 30% of the patients might have had a dysplastic process led by other HR-HPV types, especially the most common genotypes 31, 33, 35, 45, 52, and 58 [27,28]. The coexistence of more than one HR-HPV shows a 13-fold increased risk of invasive cervical cancer [29]. Therefore, the role of other genotypes should not be underestimated. Individual risk classification, treatment choices, epidemiological research, vaccine development, and preventative strategies for HPV infection are all aided by the genotyping of other HR-HPVs [30].

It should be noted that some studies show how an increase in the β1/CD29 integrin leads to the migration of basal membrane stem cells and their start of the cell cycle, promoting proliferation and migration of the cell as well as the transmission of cellular signs and, consequently, tumorigenesis [26].

The β1/CD29 integrin activates cytokines and growth factor receptors (GFRs), so the growth and invasion of cancer probably depend on the crosstalk between integrins and/or oncogenes made by cancer cells and cancer-associated cells [31].

Song et al. found that β1/CD29 integrin expression during the S phase of a cell cycle is significantly higher than its expression during the G1 phase. This finding suggests that β1/CD29 expression can promote cell proliferation [32].

The increased binding of the β1/CD29 integrin to its ligand in the extracellular matrix can disrupt different ways of cell signaling, including p53 and EGF, which leads to abnormal cell proliferation and influences the control of growth and cell differentiation [33,34]. In breast cancer patients, the β1/CD29 integrin increases radiation resistance and forms a new co-receptor complex with CD146, a distinct inducer of the epithelial-to-mesenchymal transition (EMT) that is especially strongly expressed in triple-negative breast cancer (TNBC) [35]. When both CD146 and β1 integrin activity were inhibited simultaneously, the growth of breast cancer tumors was more strongly inhibited and their susceptibility to radiation therapy was boosted [35].

In the evolutionary history of squamous lesions of cervical cells, these could be the conditions shifting the future use of the β1/CD29 integrin as a cellular marker of prognosis related to the highest risk of progression towards micro-invasive cervical cancer. Beyond squamous carcinoma, it should be remarked that adenocarcinoma accounts for approximately 20 to 25% of all cervical malignancies. The expression pattern of selected genes could be helpful prognostic factors. To this purpose, squamous cell carcinoma expresses programmed death-ligand 1 (PD-L1) more commonly than adenocarcinoma [36]. In women with squamous cell carcinoma, diffuse PD-L1 expression is linked to a much worse prognosis than marginal PD-L1 expression, which is linked to poor disease-free survival and disease-specific survival. For patients with adenocarcinomas, there is a survival advantage if the tumor does not contain tumor-associated macrophages that are positive for PD-L1 [37]. These results support the rationale for the therapeutic targeting of the PD-1/PD-L1 pathway and highlight the critical role that PD-L1 plays in the immunological escape of cervical cancer [37].

Moreover, pregnant women with intraepithelial cervical lesions up to 6–12 weeks post-partum could benefit from strict surveillance after testing positive for CD29. In this gestational age, the actual guidelines suggest postponing the evaluation and/or possible treatment if the result of a colposcopy with a biopsy rules out stromal invasion. For these women, it could be useful to have a triage test revealing the presence and level of expression of an accurate prognostic marker that identifies highest-risk cases that could develop a micro-invasive cervical carcinoma and then intensify clinic-instrumental surveillance [38,39].

For many years, the mainstay of cervical cancer prevention has been cytology-based screening. Recent European guidelines strongly recommend primary HPV-based screening over standard cytology-based screening, citing extensive evidence of higher sensitivity and accuracy, lower variability, and better reproducibility of HPV-based screening compared with conventional or liquid-based cytology [40]. Furthermore, women who have negative screening results can extend their screening intervals and self-sample using HPV-based screening [41]. The Netherlands and Turkey are the only European nations with fully operational national HPV-based cervical cancer screening programs as of July 2019. Several other nations are in various phases of implementing HPV-based screening, while Finland, Sweden, Italy, and other regions have already done so. While some nations are grappling with the inadequate efficacy of their current population-based programs, others are contemplating making the switch from cytology-based to HPV-based screening [41]. The use of HPV-based screening has increased the number of patients referred for colposcopy examinations, but it has also increased the number of CIN3+ lesions and cervical malignancies that need to be treated right once. Cytology is primarily utilized as a triage test, while, in certain nations, alternative approaches are being considered [40,42]. As many European nations, particularly those with inadequate opportunistic cytology-based cervical cancer screening programs, require immediate attention, finding additional prognostic factors should increase the sensitivity in finding high-risk patients alongside the increase in colposcopic examinations.

Of note, several limitations should be addressed in our study. The integrity of the antigen and its immune detection may have been impacted by tissue sample conditions, such as ischemia brought on by the timing of sample delivery and scarce biological sample sources during biopsies. The co-expression studies were thus constrained. Furthermore, in our experimental flow cytometry setup, we were unable to resolve non-specific binding issues since no viability dye was used in any of the running samples. This significant shortfall was solely related to the cells’ accessibility following enzymatic digestion processes. In addition, since our study provided initial insights into the issue, it could have benefited from additional evidence to increase its robustness. Lastly, it should be remarked that we did not use the latest Bethesda classification of genital tract cytology terminology, which includes both CIN2 and CIN3 in high-grade squamous intraepithelial lesions (H-SILs) in order to avoid any protocol deviation and better demonstrate the difference in the expression of the β1/CD29 integrin during the preneoplastic path. An additional limitation is the lack of cytofluorimetry and immunohistochemical analysis regarding CD29 on lesions developed due to other subtypes of HR-HPV since our protocol involved only HPV 16 and 18. Analyzing the significance of CD29 in the cervical dysplastic path led by other HR-HPVs would strengthen the robustness of the available evidence and should be carried out in additional studies.

On the contrary, to date, this study is the first one to point out the role of CD29 in HPV-associated lesions of the cervix, increasing the necessity for more clinical studies on the topic.

## 5. Conclusions

Our study shows that the β1/CD29 integrin could be used as a promising screening tool to identify the risk of developing high-grade cervical lesions. In particular, the clinical management of women belonging to high-risk classes (persistent HR-HPV infection associated with atypical squamous cells of undetermined significance and negative colposcopy, persistent HR-HPV infection, histological diagnosis of CIN1, follow-up for women already treated for high-grade lesions) might benefit from such improvement.

However, due to the acknowledged limitations, additional evidence is needed to strengthen the validity of our findings.

## Figures and Tables

**Table 1:** Immunohistochemistry analysis.

	Total (N)	% (n/N) Negative Samples	% (n/N) Samples 1+ (Weak Intensity)	% (n/N) Samples 2+ (Medium Intensity)	% (n/N) Samples 3+ (High Intensity)
Group A (CIN1)	55	65.5% (36/55)	12.7% (7/55)	12.7% (7/55)	9.1% (5/55)
Group B (CIN2)	27	33.3% (9/27)	11.1% (3/27)	22.3% (6/27)	33.3% (9/27)
Group C (CIN3)	30	13.3% (4/30)	13.3% (4/30)	40% (12/30)	33.4% (10/30)
Group D (squamous metaplasia with HPV+)	42	83.4% (35/42)	12% (5/42)	4.8% (2/42)	0

## Data Availability

The data underlying this article are available from the corresponding author upon reasonable request.
